# Eye Movements during Visuomotor Adaptation Represent Only Part of the Explicit Learning

**DOI:** 10.1523/ENEURO.0308-19.2019

**Published:** 2019-12-16

**Authors:** Zohar Bromberg, Opher Donchin, Shlomi Haar

**Affiliations:** 1 Department of Biomedical Engineering, Ben-Gurion University of the Negev, Beer-Sheva, 8410501 Israel; 2 Zlotowski Center for Neuroscience, Ben-Gurion University of the Negev, Beer-Sheva, 8410501 Israel; 3 Department of Bioengineering, Imperial College London, London SW7 2AZ, United Kingdom

**Keywords:** visuomotor rotation, explicit learning, eye movement, motor control, motor learning, motor adaptation

## Abstract

Visuomotor rotations are learned through a combination of explicit strategy and implicit recalibration. However, measuring the relative contribution of each remains a challenge and the possibility of multiple explicit and implicit components complicates the issue. Recent interest has focused on the possibility that eye movements reflects explicit strategy. Here we compared eye movements during adaptation to two accepted measures of explicit learning: verbal report and the exclusion test. We found that while reporting, all subjects showed a match among all three measures. However, when subjects did not report their intention, the eye movements of some subjects suggested less explicit adaptation than what was measured in an exclusion test. Interestingly, subjects whose eye movements did match their exclusion could be clustered into the following two subgroups: fully implicit learners showing no evidence of explicit adaptation and explicit learners with little implicit adaptation. Subjects showing a mix of both explicit and implicit adaptation were also those where eye movements showed less explicit adaptation than did exclusion. Thus, our results support the idea of multiple components of explicit learning as only part of the explicit learning is reflected in the eye movements. Individual subjects may use explicit components that are reflected in the eyes or those that are not or some mixture of the two. Analysis of reaction times suggests that the explicit components reflected in the eye movements involve longer reaction times. This component, according to recent literature, may be related to mental rotation.

## Significance Statement

Visuomotor adaptation involves both explicit and implicit components: aware reaiming and unaware error correction. Recent studies suggest that eye movements could be used to capture the explicit component, a method that would have significant advantages over other approaches. We show that eye movements capture only one component of explicit adaptation. This component scales with reaction time while the component unrelated to eye movements does not. Our finding has obvious practical implications for the use of eye movements as a proxy for explicit learning. However, our results also corroborate recent findings suggesting the existence of multiple explicit components, and, specifically, their decomposition into components correlated with reaction time and components that are not.

## Introduction

Visuomotor adaptation is commonly used to study human motor learning in health (Krakauer et al., 1999; Ghilardi et al., 2000; Taylor and Ivry, 2013; Galea et al., 2015; Haar et al., 2015b) and disease (Rabe et al., 2009; Wong et al., 2019). In a visuomotor rotation task, the visual representation of hand position is manipulated such that subjects must learn a new mapping of motor commands to apparent outcomes. Recent studies dissociated explicit and implicit processes in the visuomotor adaptation (Mazzoni and Krakauer, 2006; Hegele and Heuer, 2010; Taylor and Ivry, 2011; Taylor et al., 2014; Werner et al., 2015), where the sum of the two gives the total adaptation.

One measure of implicit learning is to ask subjects to reach straight for the target without perturbation (or without any visual feedback) and to measure the difference between the direction of reach and the target. We call this exclusion because the subject is being asked to “exclude” their explicit knowledge from their behavior. When measured after adaptation, this is called aftereffect. During adaptation, it is sometimes called a “catch trial” (Werner et al., 2015). Exclusion cannot be measured every trial since it presumes surrounding adaptation trials. To assess implicit and explicit learning throughout the adaptation process, Taylor et al. (2014) suggested simply asking subjects to report aiming direction before each movement by reporting which of the numbers displayed in a circle on the screen was in the direction the subject intended to move. Reporting has been a very productive experimental approach. However, the protocol has known limitations [e.g., reporting increases the length and variability of reaction time (RT) since subjects can start moving only after reporting].

One alternative is to measure explicit learning using eye movements: perhaps eye movements can provide an objective measure of subjects’ intentions without needing special trials or direct questioning. During unperturbed reaching movements, the eyes were found to provide an unbiased estimate of hand position (Ariff et al., 2002). During visuomotor rotation, there is an increase in correlation between gaze and hand directions in early practice, which gradually decreased thereafter (Rand and Rentsch, 2016). Indeed, a recent study found that gaze patterns during visuomotor adaptation were linked to explicit learning (de Brouwer et al., 2018). Interestingly, de Brouwer et al. (2018) noticed subjects whose eye movements did not reflect adaptation while their aftereffects did indicate some explicit learning. This raises the possibility that some forms of explicit adaptation are captured by the eye movements, while others are not.

This possibility is in line with recent suggestions of multiple explicit strategies in human motor learning, even in a redundant visuomotor rotation task (McDougle and Taylor, 2019). McDougle and Taylor (2019) showed that subjects in different conditions may use either discrete response caching or parametric mental rotation as two different explicit strategies. Their results further suggest that RT can be used to dissociate these explicit strategies: mental rotation is a time-consuming computation, and caching is a fast automatic process that does not require a long RT (Haith and Krakauer, 2018). Here, we explore the explicit components captured by eye movements and their link to the explicit strategies captured by RT.

In the first experiment of the current study, we measured subjects’ eye movements during visuomotor rotation with verbal reporting and without. As in de Brouwer et al. (2018), our results demonstrate that, in verbal reporting, eye fixations before movement onset accurately predict the reported aiming direction. Without reporting, eye fixation before movement onset correlates well with explicit learning measured by aftereffect. However, it does not account for the full explicit knowledge revealed by exclusion. This suggests that only a component of explicit learning is being captured by eye movements when there is no verbal report.

In a second experiment, we explored the time course of the discrepancy between eye movements and exclusion by introducing exclusion (catch) trials, during adaptation, in addition to testing for an aftereffect at the end of adaptation. For some subjects, measures of explicit learning from eye movements matched those from exclusion. For other subjects, exclusion revealed more explicit knowledge than that found in the eye movements. The first group was divided into the following two subgroups: those using primarily an explicit strategy and those with hardly any contribution from an explicit strategy. The second group, where exclusion showed more explicit knowledge than did the eye movements, showed subjects with the full range of combinations of explicit and implicit learning. Further analysis of RT seems to indicate that the explicit knowledge reflected in the eye movements may be the same mental rotation component identified by McDougle and Taylor (2019).

## Materials and Methods

### Participants

One hundred fourteen right-handed subjects with normal or corrected-to-normal vision participated in the study: 44 subjects (31 females; age, 18–29 years) in the first experiment and 70 subjects (46 females; age, 18–31 years) in the second experiment. Of these, five subjects were excluded due to sparse eye movement data (four subjects from the first experiment and one subject from the second), and three subjects were excluded from the second experiment because they misunderstood the exclusion instructions (details below). All subjects signed an informed consent form, which also asked for basic information about their relevant medical status. None of the subjects reported neurologic or motor impairments. The experimental protocol was approved by the Human Subject Research Committee of Ben-Gurion University of the Negev.

### Apparatus

Participants were comfortably seated on a height-adjustable chair at a table with a digitizing Wacom Intuos Pro tablet with an active area of 311 × 216 mm and a polling rate of 2154 Hz. A box was used to hide a subject’s hand and the tablet as they faced a 19 inch vertical computer screen. Their eye movements were recorded with an EyeLink 1000 (SR Research) eye-tracking system, with sampling rate of 500 Hz and the accuracy of ∼0.5°. The participants rested their head on a stabilizing support which included braces for the chin and forehead. Calibration of the eye-tracking system was performed for each subject before the experiment. During the experiment, subjects made center–out, horizontal reaching movements on the surface of the tablet using a digital stylus. On the monitor, they saw a cursor and targets to move to (detailed in the next section). The movements of the cursor matched the movements of the tablet pen (except as detailed in the next section). The EyeLink system provides the location of the pupil, and the timing and location of events including blinks, fixations, and saccades. In the reporting group (first experiment), the number that subjects reported as their intended aiming direction was recorded and typed into an Excel spreadsheet (details below).

### Trial structure

**Figure 1.**
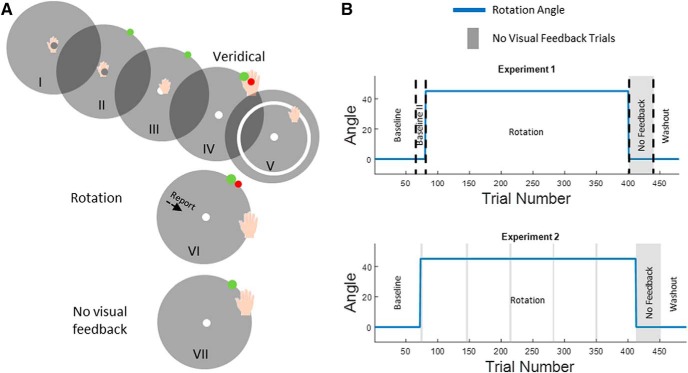
Trial and experiment design. ***A***, Basic trial structure. Subjects performed reaching movements from the origin (I). After 1 s, a target appeared (II), and the subject started reaching toward the target (III). On veridical trials, when reaching to target, a red circle appeared indicating the hand position (IV). After the feedback disappeared, a white ring appeared, which directed the hand back to the origin (V). In rotation trials, the red circle appeared rotated 45° counterclockwise (VI). In the report condition, subjects were asked to report before initiating their hand movement. In no visual feedback trials, subjects received no feedback at the end of the movement (VII). ***B***, Experimental design. Top, First experiment. In the first and second baseline block, feedback was veridical. In the rotation block, the cursor was rotated by 45°. In the no visual feedback block, the landmarks and cursor feedback were removed, and participants were instructed to aim directly at the target. In the washout block, conditions were similar to the first baseline block. In the second baseline block and in the rotation block, participants in the R condition reported their aiming direction. Bottom, Second experiment. Similar to the first experiment, however, without the second baseline block and without report sessions. In addition, five mini exclusion blocks were spread throughout the rotation block.

We used three different trial types in our experiments. The general structure of these trial types is shown in Figure 1*A*. At the beginning of every trial, subjects moved a gray cursor to a white origin. The origin appeared in the center of the screen and corresponded to a hand position at the center of the tablet. After maintaining this position for 1000 ms, a green target circle appeared at a distance of 8 cm on the screen, corresponding to a movement of 5.5 cm on the tablet (generally in the text, we will report distances on the screen and equivalent distances on the tablet will always be 0.6875 of screen distances). Visual landmarks also appeared surrounding the target. Targets could be in one of the eight cardinal and intercardinal directions relative to the origin. The origin and the target sizes on the screen were 1 and 0.5 cm^2^, respectively. The order of targets was pseudorandom for each subject and between subjects, such that a complete circuit of the targets was completed every eight movements. The cursor disappeared when the participant’s hand was further than 0.56 cm from the center of the origin. When the hand crossed the distance of 7.6 cm (95% of the target distance) from the origin, a red circle the same size as the original cursor appeared on the screen at the same distance as the target. This circle was presented to give subjects visual feedback about the cursor position at the end of the trial. The red circle that indicated the cursor location remained visible for 350, 700, or 1000 ms. Different presentation times were used in different groups and in different experiments (detailed below). After the red circle disappeared, a white ring appeared centered at the origin with radius equal to the distance of the hand from the origin. This ring guided the hand back to the origin without providing information about its exact location.

The three different trial types were as follows: veridical feedback trials where the red circle appearing at the end of each trial reflected the true location of the hand; rotation trials where the red circle appeared at a location that was rotated by 45° counterclockwise relative to the direction of hand movement; and no visual feedback trials where no red circle or any other landmarks appeared at all. In no visual feedback trials, subjects were instructed to aim straight toward the target. In all trials, subjects received auditory feedback to control movement speed: movements that reached a distance of 7.6 cm from the origin center within 500 ms were rewarded with a pleasant “ding” sound; otherwise, subjects heard an unpleasant “buzz” sound.

### Data collection

The *x* and *y* coordinates of the hand trajectories were collected by the tablet and saved from the moment of target appearance until the hand reached a distance of 7.6 cm from the origin. Eye movements were recorded continuously using the eye tracker. Eye movement data were preprocessed to remove blinks (as recorded by the eye-tracker software). In addition, eye movements were recentered to correct for drift over the experiment by assuming that the eye is fixated on the origin during the 1000 ms before the hand movement begins. Recentering was accomplished by accumulating eye position in this time window during the current trial, two preceding trials, and two following trials, and taking the median position across the entire 5000 ms of data.

### Experiment 1

#### Groups

This experiment had two groups of 22 subjects each: Report and No-Report groups. Each subject was assigned to one of two feedback times: 350 or 700 ms, counterbalanced between the groups. The feedback time showed no effect on task performance, learning, and eye movements in either of the groups, and thus data from both feedback times is combined throughout the article.

#### Procedure

Each trial presented one of the eight targets in cardinal and intercardinal directions (0°, 45°, 90°, 135°, 180°, 225°, 270°, and 315°) relative to the origin. For subjects in the Report group, 63 numbered landmarks spaced by 5.625° appeared on a circle with a radius 8 cm around the origin (the same distance from the origin as the target). Low numbers were nearer the target. Before each movement, subjects were instructed to say out loud the number toward which they were aiming to get the cursor to the target. For subjects in the No-Report group, hollow circles were presented instead of the numbered landmarks, and they were not asked to report their intended aiming direction. Indeed, the No-Report group was not informed in any way that they might want to aim to a direction different from the target. The experimental sequence was the same for both groups. Each session was divided into five blocks: two baseline blocks (72 and 8 trials) consisting of veridical trials. The first baseline (veridical feedback) block allowed subjects to get familiar with the reaching task, and the second block was intended for subjects in the Report group to practice the report. For subjects in the No-Report group, there was no difference between these two blocks. In the third block, the rotation block (320 rotation trials), the cursor was rotated relative to the origin. Subjects in the Report group were required to report their aiming direction during this block. The fourth block was a no-feedback block (40 trials), which consisted of no visual feedback trials. In the last block, a washout block (40 trials), subjects were presented with veridical feedback trials (Fig. 1*B*). The percentage of successful trials was displayed at the end of each block. A trial was considered successful if the red circle was within 5° of the target. Every 40 trials, the experimental program displayed a full-screen text message reminding subjects of the instructions.

### Experiment 2

#### Procedure

Each trial presented one of the eight targets in secondary intercardinal directions (22.5°, 67.5°, 112.5°, 157.5°, 202.5°, 247.5°, 292.5°, and 337.5°) relative to the origin. In this experiment, trial feedback was visible for 1000 ms. Forty-seven hollow circles spaced 7.5° apart appeared on the target circle as landmarks surrounding the target before each movement. The experiment was divided into the following four blocks: a baseline block with 72 veridical feedback trials, a rotation block with 320 rotation trials, a no feedback block with 40 no visual feedback trials, and the last block, a washout block with 40 veridical feedback trials. Subjects did 20 additional no visual feedback trials during the rotation block (Fig. 1*B*). These were evenly spaced during the block in five mini-blocks of four trials each. Subjects were instructed at the beginning of the experiment that their goal is to hit the target with the cursor. All instructions that were given during the experiment were presented on the screen. After the first two trials of the rotation block, a message appeared on the screen asking the subject to pay attention to the error and to hit the target with the cursor. In addition, before and after each mini-block of no visual feedback trials, a message appeared announcing the beginning and end of this block. In the beginning, the message instructed subjects to ignore their strategy and to hit the target. In the end, the message instructed them to go back to using their strategy.

### General data analysis and statistics

#### Hand movement analysis

The Hand–Target difference was calculated as the difference between the target location and the hand position when the cursor reached a distance of 7.6 cm from the origin. Trials in which the movement from the origin toward the target was not strictly increasing after the cursor passed a distance of 0.4 cm from the origin or trials in which movement was too slow were excluded. In the first experiment, subjects were presented with text reminding them of the instructions every 40 trials. Trials immediately following these reminders were discarded, since subjects often tested the degree of rotation by aiming directly at the target. Each movement RT was defined as the time between target appearance and the cursor reaching 7% of the target distance.

#### Eye movement analysis

**Figure 2.**
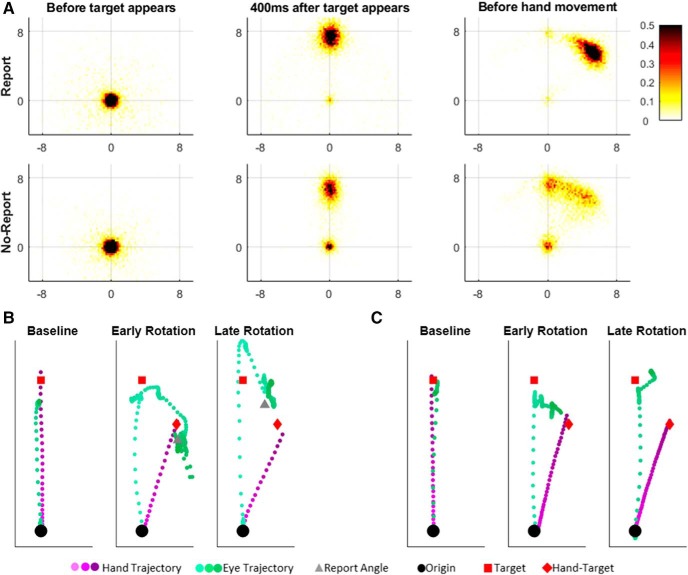
Eye movements pattern. ***A***, Fixations distributions during the phases of the trial before movement initiation, in rotation trials: the last fixation before target appears, the point of fixation 400 ms after, and the last fixation before movement initiation. Report group on top row and no-report on bottom row. Numbers on the axis indicate the distance in centimeters on the screen, 8 cm from the origin to the targets. ***B***, ***C***, Hand and eye trajectories of two individuals from the report (***B***) and the no-report (***C***) groups. In the baseline block, the subjects first gazed toward the target and later made a reaching movement toward it. At the beginning of the rotation block, both subjects shifted their gaze toward the target and later toward the Hand–Target, the reporting subject then reported a direction close to the Hand–Target, and both subjects moved their hand toward the Hand–Target. By the end of the rotation, the subjects also shifted their gaze first to the target, then toward the side opposite of the rotation. However, this secondary shift was smaller than the Hand–Target angle for the reporter and even smaller for the nonreporter. The reporter also reported a smaller angle. Both kept moving their hand to the Hand–Target direction.

The eye movements before movement initiation followed a stereotypical pattern, as follows: during the baseline block, subjects fixated first on the origin and then on the target. During the rotation block, target fixation was often followed by eye movements that carried the gaze in the direction opposite to the rotation (Fig. 2). We tested several measures of this latter gaze shift to see which best correlated with subjects’ reported aiming direction. All methods produced similar results and the choice of measure did not influence our findings. Thus, following previous results showing the eye leading upcoming hand movements with a similar constant lead (Ariff et al., 2002), we choose to use the last fixation before movement onset to characterize subjects’ intended aiming direction and called it the “explicit eye.” If eye fixation before movement onset was missing (due to a blink) or near the origin (closer than 4 cm) or beyond the target area (farther than 12 cm from the origin), then eye movements for that trial were discarded. Any subject for whom more than half of the rotation trials were discarded was excluded from further analysis (four subjects in the No-Report group in the first experiment and one subject in the second experiment were excluded because too much eye data were discarded). We used the term “implicit eye” for the difference between the explicit eye and the Hand–Target difference; it represents the estimated implicit adaptation derived using the eye movements (Table 1).

**Table 1:** Glossary

Hand–Target difference	Angular difference between the target and the hand at the end of the reaching
Explicit report	Reported direction of movement
Implicit report	Hand–Target difference minus explicit report
Explicit eye	The angle between the target and the last fixation before movement onset
Implicit eye	Hand–Target difference minus explicit eye
Implicit exclusion	The Hand–Target difference during exclusion trials
Explicit exclusion	Hand–Target difference before catch trials minus implicit exclusion

#### Reporting analysis

Report trials (in the first experiment) were also characterized by the subject’s statement of the intended aim direction. The reported number was multiplied by 5.625°/landmark to convert it to an aiming angle. This was called the “explicit report.” The “implicit report” was calculated by subtracting the explicit report from the Hand–Target difference. This is the difference between where subjects said they aim and where they moved their hand (Table 1).

#### No visual feedback trials analysis

In the no visual feedback trials, subjects were instructed to aim directly toward the target. Thus, the difference between the hand and the target in these trials represents, by definition, residual implicit knowledge of the rotation not under the subject’s control. In the second experiment, there were 20 no visual feedback trials in mini-blocks during the rotation block as well as a no visual feedback block after the rotation block. The mini-block trials and the first four trials of the no visual feedback block were named “implicit no visual feedback.” We excluded three subjects who later reported they did not understand the instruction during those trials.

#### Statistics

Unless otherwise noted, reported values are mean and SEM. A nonparametric bootstrap with 10,000 samples was used to generate the sampling distribution for group means. The 5th and 95th percentiles of the sampling distribution were used to determine the confidence intervals reported in the Extended Data. We forego reporting statistical significance as per the recommendations from Amrhein et al. (2019). Calculations of traditional hypothesis tests and effect sizes can also be found in the Extended Data.

10.1523/ENEURO.0308-19.2019.ed1 Extended Data Extended Data tables present the statistical analysis of all experimental data. The means (μ), standard deviations (σ), group differences (Δ), and effect sizes were computed on the original Data.
The 95% confidence intervals and probability to reject null hypothesis were computed on a nonparametric bootstrap with 10,000 samples. Download Extended Data, DOCX file.

The measures defined for each subject and each trial were the difference between the hand and the target (Hand–Target difference), the last fixation before movement onset (explicit eye), and the RT. In the first experiment, the report group also has the reported angle of the aim direction (explicit report). For each measure, we smoothed the results by averaging over the bins of eight sequential trials (one for each target). For each bin, trials that were more than two SDs from the mean of the bin were removed (in the first experiment, 2.0% of the available Hand–Target difference trials were outliers and 2.3% from the available explicit eye trials; in the second experiments, 1.9% and 2.3% of trials were removed, respectively). We then calculated the implicit measures for each trial by subtracting the corresponding explicit measures (eye and report) from the Hand–Target difference to create the implicit eye and implicit report. In case of a missing value in an explicit measure (due to outliers, slow movements, blinking, missed report, or other reason), no implicit measure was calculated for that measure for that trial. For each measure, we used a parametric bootstrap to find the sampling distribution of the mean for each bin, resampling data from a normal distribution with the outlier-corrected mean and SD determined by the eight points in each bin for each measure (or fewer points where there were missing values).

In the second experiment, we also measured “implicit exclusion,” the Hand–Target difference during exclusion trials. We averaged over the four sequential trials of each mini-block to create a binned version of implicit exclusion, and again used a parametric bootstrap to find the sampling distribution of the mean of each bin, resampling data from a normal distribution with mean and SD determined by the four points in each bin. Similarly, we also averaged the Hand–Target difference, the explicit eye, and the implicit eye in the four trials preceding each mini exclusion block. We used these to calculate the “explicit exclusion” by subtracting the binned implicit exclusion from the binned Hand–Target difference before the mini exclusion block (Table 1). We defined the “implicit difference” as the difference between the binned implicit eye before the mini exclusion block and the implicit exclusion. Since, for some subjects, some bins were missing, we used probabilistic principal components analysis (PPCA) (Tipping and Bishop, 1999) to fill in the missing values by projecting the data (6 bins/subject) onto the first five principal components (PCs) space and then back to the original 6-dimensional space.

In the reaction time analysis, we binned the Hand–Target difference and the explicit eye according to the RT. We divided the reaction time into windows of 25 ms from 0 to 2000 ms and averaged over the Hand–Target difference and the explicit eye of all trials with RT in the same window. The SEM for each window was calculated by dividing the SD of each bin by the square root of the number of trials in this bin.

In all learning curves, we focused on three phases: the “initial rise” was characterized by the third bin in the rotation block (trial numbers 97–104; rotation trials 17–24); the “late early rise” was characterized by the 10th bin in the rotation block (trial numbers 153–160; rotation trials 73–80), and the “end of adaptation” was characterized by the last bin of the rotation block (trial numbers 393–400; rotation trials 313–320).

#### Clustering

In the second experiment, we clustered the subjects with fuzzy c-means (FCM) clustering. We tested clustering for two through six clusters. Following Haar et al. (2015a), we used the cluster validity index proposed by Zhang et al. (2008). This index uses a ratio between a variation measure in each cluster and a separation measure between the fuzzy clusters. The smaller the ratio, the better the clustering. Clustering was applied in two steps: the first on the six binned values of implicit difference; the second on the explicit eye during rotation trials. For each cluster found in the first step, perform additional clustering on the first three PCs from the 40 bins of rotation trials.

## Results

We recorded eye movements that subjects made during hand-reaching movements perturbed by a clockwise visuomotor rotation to study the relation between subjects’ gaze and explicit learning. In the first experiment, we developed and validated our explicit eye measure, and, in the second experiment, we explored the time course of the explicit eye measure and its relation to exclusion, a measure of implicit learning.

### Experiment 1

**Figure 3.**
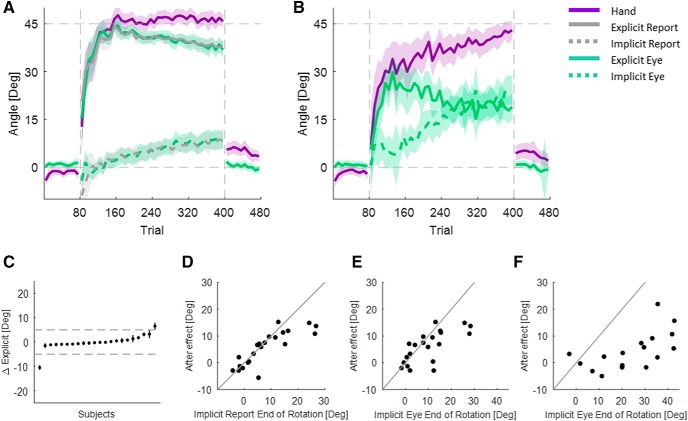
Experiment 1. ***A***, Learning curves of the report group. ***B***, Learning curves of the no-report group. ***C***, Subjects’ differences between the explicit report and the explicit eye. Each dot is the averaged difference between the explicit report and the explicit eye during the rotation block, and each line shows the SEM of this difference. Dashed gray lines show a 5° region of equivalence. ***D***, The relation between the aftereffect and the implicit report at the end of adaptation in the report group. ***E***, The relation between the aftereffect and the implicit eye at the end of adaptation in the report group. ***F***, The relation between the aftereffect and the implicit eye at the end of adaptation in the no-report group. ***D–F***, Dots denote individual subjects; identity line is in black.

The first experiment followed the protocol of Taylor et al. (2014) and compared a group adapting while reporting (R) with one that did not report (NR). Both groups showed an initial rapid rise in the Hand–Target difference (Fig. 3*A*,*B*). The initial rise was 32.9 ± 6.2° and 24.7 ± 8.5° for the R and NR groups, respectively. The difference between the groups in the initial rise was 8.2 ± 10.5°. After the initial rise, adaptation continues albeit more slowly. This slower rise continues until subjects have zero error. The report group plateaued after ∼80 trials with mean Hand–Target difference of 46.5 ± 2.8° at the late early rise. The NR group was slower to reach plateau and at the late early rise their mean Hand–Target difference was only 33.9 ± 5.4°. The difference between groups in this late early adaptation phase is 12.6 ± 6.1°. By the end of adaptation phase, the mean Hand–Target difference is 45.8 ± 2.1° and 43.0 ± 2.7° in the R and NR groups, respectively.

Explicit report and explicit eye in both groups rise quickly and fall off slowly, reflecting a slow but steady increase in implicit knowledge. Differences between groups in the explicit eye at the initial rise (difference, 15.8 ± 8.6°) and in the late early rise (difference, 19.5 ± 6.0°) mirrors the difference in the Hand–Target difference, reflecting the dominant role of explicit knowledge in initial learning across both groups. By the end of adaptation, the difference between the R and NR groups still exists (18.0 ± 4.8°).

In the R group, explicit eye and explicit report match with an average difference of −0.02 ± 0.8°. This is true not only on average, but also for each subject (Fig. 3*C*). As a consequence, the corresponding implicit measures also match. The average difference is −0.3 ± 0.8°, and this is consistent with the aftereffect measure of the implicit: the difference between the aftereffects and the implicit components is 3.4 ± 3.0° (Fig. 3*D*,*E*). This contrasts with the consistent lack of a match between implicit eye and aftereffect in the NR group (Fig. 3*F*). For the NR group, the implicit eye measure suggested a much larger implicit component than revealed by the aftereffect, where the difference between the aftereffects and the implicit eye was 19.6 ± 4.5°. Nevertheless, the aftereffect and the implicit eye correlated in both groups, with mean Spearman correlations of 0.75 ± 0.09 and 0.67 ± 0.11 for the R and NR groups, respectively. This suggests the possibility of a shared component, despite the difference in average values.

### Experiment 2

To explore the difference between eye movements and the aftereffect, we conducted a second experiment where we added exclusion trials during the rotation block. Those trials were no visual feedback trials in which we asked subjects to ignore their strategy and aim directly at the target. Since, by aiming at the target, they remove the expression of their explicit knowledge, the remaining Hand–Target difference reflects only their implicit learning. Five mini-blocks of four exclusion trials were used during the rotation, and these were combined with a virtual mini-block of the first four exclusion trials of the aftereffect. This led to a total of 24 exclusion trials in six mini-blocks for four trials each.

**Figure 4.**
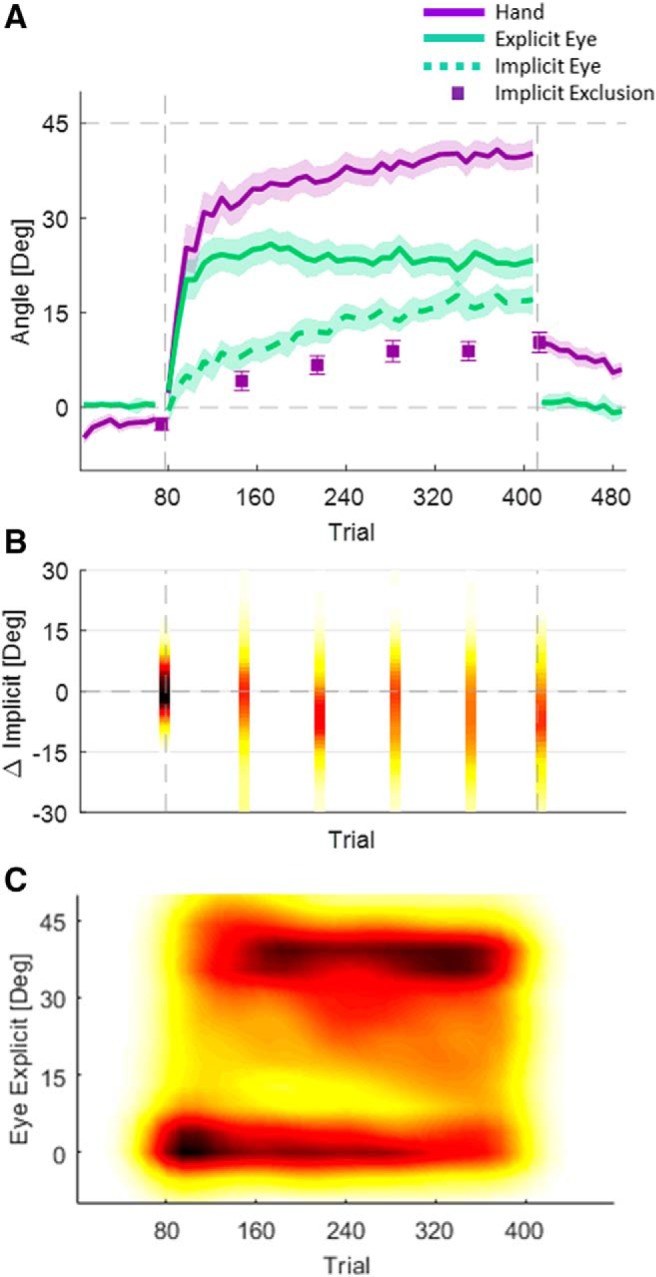
Experiment 2. ***A***, Averaged learning curves across the entire group. Error bars in the implicit exclusion and the shaded area represent the SEM. ***B***, Distribution of the differences between the two implicit measures. ***C***, Distribution of the explicit eye.

As in the NR group of the first experiment, the Hand–Target difference initially rose quickly, and later continued to rise slowly (Fig. 4*A*). In this experiment, on average, subjects did not reach full adaptation. Here, too, the explicit eye initially rises quickly. However, it did not decline as much as in the first experiment. The implicit eye also rose slowly, but less than in the first experiment. (This is a computational consequence of the reduction in the explicit eye mentioned above. The implicit eye is simply the difference between Hand–Target difference and explicit eye.) Replicating experiment 1, there was a gap between the implicit exclusion and the implicit eye. On average, the Hand–Target difference showed an initial rise of 25.2 ± 3.7°. By the end of adaptation, the Hand–Target difference reached 40.2 ± 2.2°. The explicit eye, on average, had an initial rise of 20.2 ± 3.1° and stabilized at this level until the end of the adaptation, where it reached 23.3 ± 2.7°. The implicit exclusion was lower than the implicit eye, with an average gap of 5.4 ± 1.3°. However, this gap was not consistent across subjects (Fig. 4*B*). For some subjects, almost no gap existed, while, for others, it was quite substantial.

Additionally, subjects who did not show a gap between implicit eye and implicit exclusion seemed to be of two types. For some, gaze stayed on the target, reflecting an explicit eye adaptation of 0° (these subjects also had a very slow rise in Hand–Target difference, which reached no more than 30°). For others, gaze shifted to the Hand–Target early in adaptation, reflecting nearly full explicit knowledge (Fig. 4*C*). Subjects in this latter group also had a very quick initial rise in the Hand–Target difference. These results suggest extensive explicit adaptation in these subjects.

**Figure 5.**
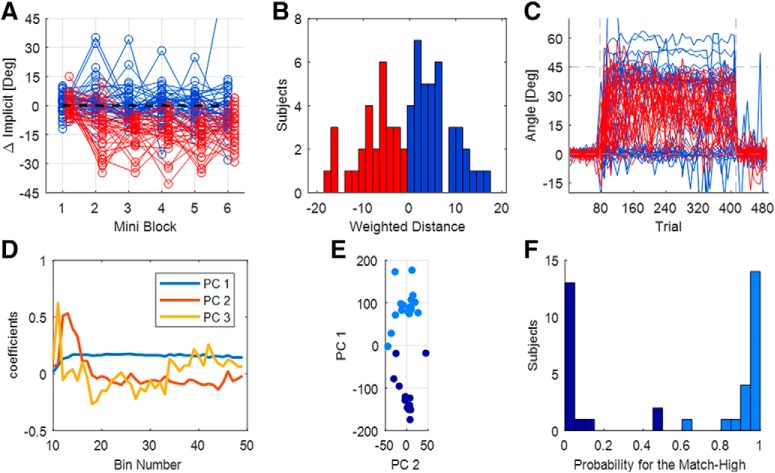
Experiment 2: clustering steps. ***A–C***, Clustering step 1. Blue lines are subjects who belong to the cluster in which the implicit measures match, and red lines are those who belong to the cluster in which the implicit measures do not match. ***A***, The difference between the two implicit measures for the six mini-blocks of exclusion. Each line depicts an individual subject. ***B***, Histogram of the weighted averaged implicit difference. ***C***, Explicit eye for all subjects. ***D–F***, Clustering step 2. ***D***, Three principal components on which the data are clustered are shown. ***E***, The first and the second PCs. Dots represent individual subjects: light blue dots are subjects with high explicit eye, and dark blue dots are subjects with low explicit eye. ***F***, Histogram of the probability of subjects to belong to the high explicit eye cluster.

Having noticed this pattern in the data, we clustered the subjects accordingly (Fig. 5). We used a two-step approach. In the first step, we clustered subjects using FCM according to the difference between the two measures of implicit. The cluster validity index (Zhang et al., 2008) values suggested two clusters. As expected, for one cluster implicit eye and implicit exclusion matched, while for the other cluster implicit exclusion was greater than implicit eye. We called these the “matched-implicit” and “No-Match” clusters. In the second clustering step, we further clustered the matched-implicit cluster according to the explicit eye measured throughout the entire rotation block. We used PPCA to reduce the dimensionality of the data. We ran FCM clustering over the first three components, which captured 93% of the overall variance. The cluster validity values again suggested two clusters. Applying the same clustering approach to the No-Match group did not reveal any evidence of clustering.

**Figure 6.**
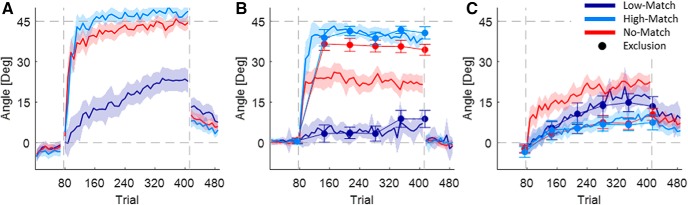
Experiment 2: learning curves per cluster. ***A***, Hand–Target difference for the Match-Low, Match-High and No-Match clusters. ***B***, Explicit eye (lines) and explicit exclusion (circles) for the three clusters. ***C***, Implicit eye (lines) and implicit exclusion (circles) for the three clusters. Error bars around the implicit exclusion and the shaded area represent the SEM.

The two-step clustering method found three clusters of subjects. Two of them are derived from the matched-implicit cluster of the first step, and, for them, the explicit eye faithfully reflects explicit knowledge. From those two, we called the one that had more explicit adaptation “Match-High” (*n* = 21). The Hand–Target difference of these subjects rose quickly to 31.8 ± 6.6° during the initial rise, and then to 45.4 ± 2.2° at the late early rise, and to 48.7 ± 2.4° at the end of adaptation (Fig. 6*A*). This group of subjects counteracts the rotation fully and quickly using an explicit strategy. This can be seen in the explicit eye, which rose quickly to 31.0 ± 6.2° and later to 41.6 ± 2.7°, and by the end of adaptation was slightly reduced to 38.8 ± 2.3° (Fig. 6*B*). Their implicit learning, measured by implicit eye, was 10.6 ± 2.8° at the end of the rotation block (Fig. 6*C*). For this group, explicit eye and implicit eye matched explicit exclusion and implicit exclusion (differences of 0.5 ± 1.1° and −0.3 ± 1.6°, respectively).

In contrast, the other matching cluster had very little explicit adaptation. We called it “Match-Low” (*n* = 17). For these subjects, the difference between explicit eye and explicit exclusion was also very small (−0.1 ± 1.9°), as was the case for implicit eye and implicit exclusion (−0.5 ± 2.4°). The lack of explicit strategy is reflected in the very low explicit eye, which was 3.0 ± 4.2° in the initial rise, 5.8 ± 4.0° at the late early rise, and reached 7.1 ± 5.9° at the end of adaptation (Fig. 6*B*). Since this cluster used no explicit strategy, the subjects adapt to the rotation only implicitly, and thus have very slow adaptation (Fig. 6*A*). The average Hand–Target difference changed from 3.6 ± 5.6° at the initial rise to 12.7 ± 4.7° at the late early rise and reached 22.7 ± 4.3° at the end of the rotation block. Hence, the Match-High and Match-Low groups had very different Hand–Target difference and explicit eye. The implicit eye of the Match-Low group reached 16.1 ± 6.5° by the end of the rotation block, 5.5 ± 7.1° higher than that of the Match-High group.

The No-Match cluster (*n* = 28; which was the second cluster in step 1) was characterized by a lack of correspondence between implicit eye and implicit exclusion (Fig. 6*C*). Accordingly, this cluster adapts to the rotation using a mostly explicit strategy (Fig. 6*B*). However, the explicit eye captures only part of the explicit learning measured with explicit exclusion. The difference between them, across subjects, is 13.5 ± 1.5° by the end of the rotation block. The fact that explicit adaptation does exist in these subjects is supported by the rapid rise of the Hand–Target difference: 33.5 ± 5.0° in the initial rise, 39.8 ± 2.8° at the late early rise, and 44.5 ± 2.5° at the end of adaptation. This performance is similar to that of the Match-High group, with differences of −1.7 ± 8.2°, 5.6 ± 3.6°, and 4.2 ± 3.5° at the initial rise, at the late early rise, and at the end of adaptation. In contrast, the explicit eye of this No-Match group was very different from that of the Match-High. It changed from 22.6 ± 4.1° in the initial rise to 24.1 ± 3.6° at the late early rise and to 21.5 ± 3.3° by the end of adaptation. Both measures of implicit of the No-Match cluster rose slowly; the implicit eye was much higher than implicit exclusion and the difference was 13.3 ± 1.7° by the end of the rotation block.

**Figure 7.**
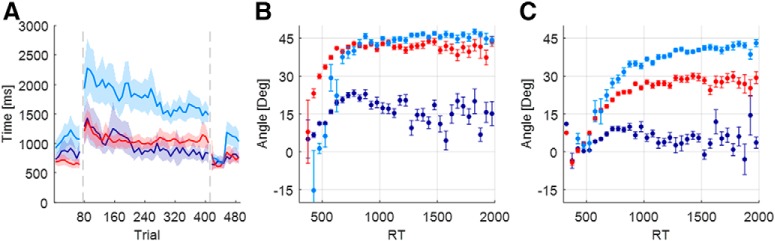
Experiment 2: reaction times. ***A***, Averaged reaction time for each cluster. The shaded area represents the SEM. ***B***, ***C***, Cluster means of Hand–Target differences (***B***) and cluster means of explicit eye as a function of RT (binned in 25 ms bins; ***C***). Each dot is a bin, and error bars represent the SEM of each bin.

Following previous studies that suggested that explicit learning requires longer RTs (Benson et al., 2011; Leow et al., 2017; McDougle and Taylor, 2019), we looked for differences in the RT between our different explicit learning groups (Fig. 7*A*). The Match-High group showed the longest RTs. Their RTs decreased by 758 ± 370ms from initial adaptation to the end of adaptation, which is in line with the decrease in their explicit eye over the adaptation period. The RTs of those in the Match-Low group, who had almost no explicit adaptation, were faster. Though the explicit eye of the Match-Low group increased during adaptation, their RTs decreased by 450 ± 341 ms. The RTs of the No-Match group started as fast as those of the Match-Low group, and they grew quicker by 272 ± 228 ms. Interestingly, the Match-High group had longer RTs in the baseline and the washout blocks as well. The RTs of this group were different from those of the low-match and the No-Match groups by 249 ± 181 and 404 ± 152 ms, respectively. It appears that behavior in the clusters is different even before adaptation begins.

Figure 7, *B* and *C*, shows the relationship between RT and Hand–Target difference and between RT and explicit eye. For RTs >400 ms, larger RTs were associated with larger Hand–Target differences and with more explicit eye. This relationship saturated in the Match-Low group for RTs longer than 700 ms. While the results are partially consistent with the hypothesis that explicit eye reflects aspects of explicit knowledge, we also expected that averaged RTs in the No-Match group would be closer to those of the Match-High group than to those of the Match-Low group. In fact, Figure 7*A* shows that the opposite is the case. For this group, the explicit eye reflects only one component of explicit learning, the same one that is correlated to the RTs, while there is another explicit component that is not related to either eye movements or RTs.

## Discussion

In this study, we explored the extent to which explicit components of visuomotor adaptation are reflected in eye movements. We did this by comparing eye movements to two accepted measures of explicit learning: verbal report and the exclusion test. Our experiments showed that eye movements have a stable pattern: after target appearance, the eyes saccade from the origin to the target, and then, before movement onset, the eyes saccade again in the direction toward which the subject will aim. We believe that these eye movements provide a measure of explicit adaptation (we called it explicit eye); however, this measure only reflects part of the explicit adaptation. Our first experiment showed that when subjects report their intended direction, explicit eye and the other two measures (verbal report and exclusion) all matched. In contrast, when subjects did not report, explicit eye only reflected part of the explicit adaptation, as reflected in the exclusion. However, the two were correlated, and this suggests that explicit eye might be reflecting select components of the explicit exclusion. In our second experiment, we tried to explore more fully the time course of the separation of explicit eye from explicit shown by exclusion. We found that the two diverge early in adaptation. In analyzing the data of the second experiment, we found three groups of subjects. The first group adapted fully to the rotation and had eye movements consistent with performance in exclusion trials (Match-High group); the second group also adapted fully but had less explicit eye than would be expected from exclusion trials (No-Match group); the third group only adapted partially and had eye movements consistent with lack of explicit adaptation in the exclusion trials (Match-Low group). The learning curves of this last group were similar to those reported in paradigms where subjects had only implicit adaptation (Morehead et al., 2017; Kim et al., 2018, 2019).

Smith et al. (2006) proposed a two-state model of motor adaptation that is still the most widely used model in the field. It has been nicely mapped onto explicit (fast) and implicit (slow) components of adaptation (McDougle et al., 2015). However, there have been suggestions that there are more than two states in the adaptation process, and that there may be multiple explicit and implicit components, potentially with different time constants. Forano and Franklin (2019) showed that dual adaptation can be best explained by models with two fast components, and McDougle and Taylor (2019) showed two explicit strategies in visuomotor rotation: caching and mental rotation. Presumably, in our study, the group for which explicit eye explained only part of the explicit learning (No-Match group) used multiple explicit strategies, while the group where measures of explicit learning matched used one (Match-High group) or, perhaps, none (Match-Low group).

The question arises whether the components of the explicit adaptation that are reflected in explicit eye map to the explicit strategies identified by McDougle and Taylor (2019). In that study, the key difference in the strategies was that one strategy introduced a correlation between rotation and reaction time while the other did not. Consequently, we examined reaction times in the different groups. We found that the group with the single explicit strategy (captured by gaze; Match-High group) had very long reaction times relative to the other groups. Interestingly, these subjects had longer reaction times in the baseline phase as well, suggesting that they were more carefully and explicitly controlled movers even during normal movement. The reaction times of the No-Match group were much lower. That is, the No-Match group achieved explicit adaptation comparable to that of the Match-High group, but their explicit adaptation required less preparation time. The Match-Low subjects, who only adapted implicitly, had the fastest reaction times. Together, we hypothesize that the explicit components reflected in explicit eye are the same components that drive longer reaction times. McDougle and Taylor (2019) identified this as the process of mental rotation and contrasted it with the low-reaction time mechanism of caching.

Links between the intended direction of movement and eye movements have been foreshadowed (Rentsch and Rand, 2014; Rand and Rentsch, 2016) and demonstrated explicitly (de Brouwer et al., 2018). Our study supports these earlier findings, although there are some technical issues that deserve consideration. First, we followed the Rand and Rentsch (2016) study in using only end-point feedback rather than continuous presentation of the cursor. This simplified the eye movements and allowed us to determine that the fixations immediately before movement initiation provided the most reliable estimate of explicit adaptation. The specific timing at which eye movements are considered has consequences. Our findings match those of de Brouwer et al. (2018) in that both studies find that eye movements reflect explicit adaptation. An important difference in the findings relates to the timing at which eye movements are considered. de Brouwer et al. (2018) evaluate the fixation closest to the rotation angle. Our data support the basic statistical logic that such a measure will tend to be biased. The last fixation before movement onset was a more stable measure and is consistent with earlier results on the specific timing with which eye movements predict hand movements (Ariff et al., 2002). This measure also allowed an unbiased quantification of explicit adaptation even in subjects with very little explicit adaptation, which is key for identifying the Match-Low group. This difference in the measures may be one reason why de Brouwer et al. (2018) did not identify the three different groups of subjects we found.

Last, we note that each of our measures (report, eye, and exclusion) measured either explicit adaptation or implicit adaptation, but not both. We then calculated the complementary adaptation by subtracting the measured component from the hand direction. Much influential research in the field takes this approach: it assumes that hand direction is the simple sum of an explicit and an implicit component (Taylor et al., 2014; Huberdeau et al., 2015; McDougle et al., 2015; Christou et al., 2016; Leow et al., 2017). However, this assumption has been questioned, and various efforts to validate it have been put forward including the use of inclusion trials in combination with exclusion trials (Werner et al., 2015; Neville and Cressman, 2018; Modchalingam et al., 2019). Exclusion trials test for explicit knowledge by asking subjects to stop using what they know. Inclusion trials verify this ability to explicitly control behavior by asking subjects to go back to using what they know. Since, in most studies, inclusion trials show less adaptation than do the rotation trials that preceded them, it seems that the total behavior must involve some component that is more easily turned off than turned back on. This is in line with the claim in this article that explicit knowledge may involve multiple components. We use inclusion trials to further explore this idea in the study by Maresch and Donchin (2019).

This study provides replicates and extends earlier findings that eye movements reflect an explicit strategy in visuomotor adaptation. It supports other reports demonstrating multiple explicit components in adaptation. It seems that some components of explicit adaptation are not reflected in the eye movements. The components reflected in the eye movements are correlated with reaction time and may include the component identified by McDougle and Taylor (2019) as mental rotation. While eye movements may not be a perfect measure of explicit adaptation, they could be used to capture this component on a trial-by-trial basis without influencing the adaptation.

## Synthesis

Reviewing Editor: Satu Palva, University of Helsinki Decisions are customarily a result of the Reviewing Editor and the peer reviewers coming together and discussing their recommendations until a consensus is reached. When revisions are invited, a fact-based synthesis statement explaining their decision and outlining what is needed to prepare a revision will be listed below. The following reviewer(s) agreed to reveal their identity: Denise Henriques, Santeri Rouhinen.

The study provides new insights about the implicit and explicit components of visuomotor learning but comparing different measures for tapping into these mechanisms. The objective was to measure different aspects of the explicit components during terminal visuomotor adaptation using a few “common” measures - gaze direction prior to reach onset, the pre-reach aiming-strategy task, and measures of exclude-strategy reach aftereffects (in a follow up experiment). The experiments are carefully conducted and analysed using non-conventional methods (as well as conventional tests in the appendix). The results indeed replicate the early finding that eye movements reflect explicit processes but suggest that some of this explicit component during adaptation is not captured by the gaze alone, given the other measures of strategy use. The results are interesting, novel, and can partly explain individual differences in adaptation performance and explicit strategy use. This is the one of the few studies that delve into individual differences in adaptation and strategy use.

Major

For the gaze measure, the authors attempt to improve upon the original paradigm by de Brouwer et al in several ways, and again combine this assay with those of other measure of explicit learning to test whether multiple explicit processes may be involved in adaptation as suggested by the recent McDongle and Taylor 2019 study.

It's too bad the authors didn't include trials where subjects were asked to implement a strategy for trials where the cursor was not visible, to complement the Exclusion reach trials, as done by Werner et al., 2015, Neville and Cressman (2017), and Modchalingam et al (2019). In those studies, the inclusion-reach aftereffects didn't fully compensate for the rotation as would be suggested by the Explicit Exclusion measure shown in this study which is just merely subtracting the Implicit Exclusion reach deviation from the Hand-Target error. This suggests that merely subtracting one measure from the other to get “indirect” measures of explicit and implicit components may not be totally justifiable, despite its common use.

Explicit Exclusion which is a bit of an unfortunate “name”. I understand Explicit Exclusion is just the Implicit Exclusion reaches minus the Hand-Target Error, and is analogous with the Explicit vs Implicit Eye. Perhaps the authors may want to remind the reader the origin of “Explicit Exclusion”.

The figures are nice and clear, but we would suggest replacing the yellow with red. It's hard to see the yellow dots especially when they are overlapping with the other groups.

The manuscript would benefit greatly from an external language revision. While parts of the text are written clearly, much of the text is difficult to read. There are several mistakes that should be corrected. Please proofread carefully.

The terminology like Explicit/Implicit eye, Implicit report are difficult to read for those outside of the field. Please clarify the text regarding to the meaning of each term. A glossary with the explanations would be helpful. For instance, implicit exclusion was not defined in the current manuscript.

Are the measured subject and trial numbers as reported before or after discards? If after, please report the numbers that were used for analyses.

It is found that the adaptation indicated by the exclusion were correlated, and it suggested that

Explicit Eye might be reflecting components of explicit adaptation (line 462). The Figure 3F is referred in this respect. Please clarify how is Figure 3F related to this finding. There seems to be no correlation in 3F.

Minor

Figure 2A doesn't have axis units.

Were the tablet and stylus visible to the subjects?

Are the bootstrap confidence intervals used only in supplementary tables? They are mentioned in the methods section, but I didn't notice them being reported in results.

Data collection. x and y coordinates of the hand trajectories were collected by the tablet and saved from the moment of target appearance until the hand reached 95% of the targets

circle radius. It would be helpful to get the target size as tablet cm2 also.

## Author Response

Major

For the gaze measure, the authors attempt to improve upon the original paradigm by de Brouwer et al in several ways, and again combine this assay with those of other measure of explicit learning to test whether multiple explicit processes may be involved in adaptation as suggested by the recent McDongle and Taylor 2019 study.

It's too bad the authors didn't include trials where subjects were asked to implement a strategy for trials where the cursor was not visible, to complement the Exclusion reach trials, as done by Werner et al., 2015, Neville and Cressman (2017), and Modchalingam et al (2019). In those studies, the inclusion-reach aftereffects didn't fully compensate for the rotation as would be suggested by the Explicit Exclusion measure shown in this study which is just merely subtracting the Implicit Exclusion reach deviation from the Hand-Target error. This suggests that merely subtracting one measure from the other to get “indirect” measures of explicit and implicit components may not be totally justifiable, despite its common use.

Explicit Exclusion which is a bit of an unfortunate “name”. I understand Explicit Exclusion is just the Implicit Exclusion reaches minus the Hand-Target Error, and is analogous with the Explicit vs Implicit Eye. Perhaps the authors may want to remind the reader the origin of “Explicit Exclusion”.

----------

Regarding the term “Explicit Exclusion”: it is indeed the subtraction between Implicit Exclusion and the Hand-Target Error and is analogous with the Explicit and Implicit Eye and Explicit and Implicit Report. In all cases, we use subtraction of the measured quantity from Hand-Target Error to calculate the complementary component. This is now clarified in the glossary table we have added.

We also agree with the referee that inclusion trials are important for interpretation and should be more widely used. As the referee notes, inclusion trials demonstrate that the arm movement is not simply a sum of an implicit and an explicit component, a fact that is often overlooked. We added the following paragraph to the discussion to emphasize this point of interpretation:

“Lastly, we note that our measures (report, eye, and exclusion) measured either explicit adaptation or implicit adaptation. We then calculated the complementary adaptation by subtracting the measured component from the hand direction. Much influential research in the field takes this approach: it assumes that hand direction is the simple sum of an explicit and implicit component (e.g. Christou et al., 2016; Huberdeau et al., 2015; Leow et al., 2017; McDougle et al., 2015; Taylor et al., 2014). However, this assumption has been questioned and various efforts to validate it have been put forward including the use of inclusion trials in combination with exclusion trials (e.g. Modchalingam et al., 2019; Neville and Cressman, 2018; Werner et al., 2015). Exclusion trials test for explicit knowledge by asking subjects to stop using what they know. Inclusion trials verify this ability to explicitly control behavior by asking subjects to go back to using what they know. Since, in most studies, inclusion trials show less adaptation than do the rotation trials that preceded them, it seems that the total behavior must involve some component that is more easily turned off than turned back on. This is in line with the claim in this paper that explicit knowledge may involve multiple components. We use inclusion trials to further explore this idea in Maresch and Donchin (2019).”

------------------------------------------------------------------------------------------------------

The figures are nice and clear, but we would suggest replacing the yellow with red. It's hard to see the yellow dots especially when they are overlapping with the other groups.

----------

Thank you for this input. We have changed the colors accordingly.

------------------------------------------------------------------------------------------------------

The manuscript would benefit greatly from an external language revision. While parts of the text are written clearly, much of the text is difficult to read. There are several mistakes that should be corrected. Please proofread carefully.

----------

The revised manuscript was carefully proofread and we hope that the reviewers find the revised text clearer.

------------------------------------------------------------------------------------------------------

The terminology like Explicit/Implicit eye, Implicit report are difficult to read for those outside of the field. Please clarify the text regarding to the meaning of each term. A glossary with the explanations would be helpful. For instance, implicit exclusion was not defined in the current manuscript.

----------

We thank the reviewer for this important input. A glossary was now added to the manuscript (table1) and implicit exclusion is now defined in the text as well (line 263 in the revised manuscript)

------------------------------------------------------------------------------------------------------

Are the measured subject and trial numbers as reported before or after discards? If after, please report the numbers that were used for analyses.

----------

The numbers in the text represent subject numbers and trial counts before exclusion. In the revised version, we report the number of excluded subjects in the ‘participants’ section of the methods, following the reported number of subjects. We hope this prevents confusion (line 97 in the revised manuscript).

------------------------------------------------------------------------------------------------------

It is found that the adaptation indicated by the exclusion were correlated, and it suggested that Explicit Eye might be reflecting components of explicit adaptation (line 462). The Figure 3F is referred in this respect. Please clarify how is Figure 3F related to this finding. There seems to be no correlation in 3F.

----------

Figure 3F does show correlation between implicit Eye and the after effect (Spearman correlation of 0.67, as reported in the text (line 329 in the revised manuscript)). However, we agree that referring to the figure at this point can cause confusion and we have removed the reference.

------------------------------------------------------------------------------------------------------

Minor

Figure 2A doesn't have axis units.

----------

Units (cm) were added to the figures caption

------------------------------------------------------------------------------------------------------

Were the tablet and stylus visible to the subjects?

----------

The tablet and stylus were not visible to the subjects, as in most visuomotor adaptation studies. We clarified it in the methods text (line 106 in the revised manuscript).

------------------------------------------------------------------------------------------------------

Are the bootstrap confidence intervals used only in supplementary tables? They are mentioned in the methods section, but I didn't notice them being reported in results.

----------

The bootstrap confidence intervals are indeed reported only in the extended data and not in the main text. We have clarified this in the methods (line 244 in the revised manuscript).

------------------------------------------------------------------------------------------------------

Data collection. x and y coordinates of the hand trajectories were collected by the tablet and saved from the moment of target appearance until the hand reached 95% of the targets

circle radius. It would be helpful to get the target size as tablet cm2 also.

----------

The target distance was 8 cm on the screen, corresponding to a movement of 5.5 cm on the tablet. Thus, all sizes and distances which are reported on the screen can be translate to the tablet with a ratio of 0.6875. We now explain it in the text (line 123 in the revised manuscript).

The target size was (0.5 cm2) on the screen, corresponding to about 0.24 cm2 on the tablet. The origin and the target sizes are now reported in the text (line 126 in the revised manuscript).
