# Supplementary material for: Eye Movements during Visuomotor Adaptation Represent Only Part of the Explicit Learning
Source: eNeuro. 2019 Dec 16;6(6):ENEURO.0308-19.2019. doi: 10.1523/ENEURO.0308-19.2019 (PMC6978919; doi:10.1523/ENEURO.0308-19.2019)
Supplement: Extended Data — tables present the statistical analysis of all experimental data. The means (μ), standard deviations (σ), group differences (Δ), and effect sizes were computed on the original Data. The 95% confidence intervals and probability to reject null hypothesis were computed on a nonparametric bootstrap with 10,000 samples. Download Extended Data, DOCX file. [file sup_enu-eN-NWR-0308-19-s02.docx]

# Extended Data

The next tables present the statistical analysis. The $\mu, \sigma,$ Δ and effect size computed on the original Data. The CI and probability to reject null computed on the sampled distribution.

Effect size is calculated either by $\frac{\mu}{\sigma}$ or by $\frac{\Delta}{\sigma}$.

**Experiment 1**

**Initial rise in Hand-Target Difference:**

| group | $\mu$ | $\sigma$ | 2.5% | 97.5% | Prob to reject null ($\Delta>0)$ | Effect size |
| --- | --- | --- | --- | --- | --- | --- |
| NR | 24.7 | 20.8 | 8.2 | 41.3 | 0.18% | 1.2 |
| R | 32.9 | 22.5 | 19.1 | 43.6 | 0% | 1.5 |

Differences between groups:

| Δ | 2.5% | 97.5% | Prob to reject null | | Effect size |
| --- | --- | --- | --- | --- | --- |
|  |  |  | $\Delta>0$ | $\Delta=0$ |  |
| 8.2 | -12.7 | 28.8 | 21% | 73% | 1.7 |

**Late early rise in Hand-Target Difference:**

| group | $\mu$ | $\sigma$ | 2.5% | 97.5% | Prob to reject null ($\Delta>0)$ | Effect size |
| --- | --- | --- | --- | --- | --- | --- |
| NR 1^st^ | 33.9 | 13.7 | 22.7 | 43.9 | 0% | 2.5 |
| R 1^st^ | 46.5 | 5.6 | 40.9 | 52.1 | 0% | 8.2 |

Differences between groups:

| Δ | 2.5% | 97.5% | Prob to reject null | | Effect size |
| --- | --- | --- | --- | --- | --- |
|  |  |  | $\Delta>0$ | $\Delta=0$ |  |
| 12.6 | 0.9 | 25.2 | 2% | 73% | 4.1 |

**End of adaptation in the Hand-Target Difference:**

| group | $\mu$ | $\sigma$ | 2.5% | 97.5% | Prob to reject null ($\Delta>0)$ | Effect size |
| --- | --- | --- | --- | --- | --- | --- |
| NR | 43.0 | 6.6 | 37.7 | 48.3 | 0% | 6.5 |
| R | 45.8 | 4.6 | 41.5 | 49.8 | 0% | 9.8 |

Differences between groups:

| Δ | 2.5% | 97.5% | Prob to reject null | | Effect size |
| --- | --- | --- | --- | --- | --- |
|  |  |  | $\Delta>0$ | $\Delta=0$ |  |
| 2.8 | -3.9 | 9.4 | 21% | 27% | 1.2 |

**Initial rise in Explicit Eye:**

| group | $\mu$ | $\sigma$ | 2.5% | 97.5% | Prob to reject null ($\Delta>0)$ | Effect size |
| --- | --- | --- | --- | --- | --- | --- |
| NR | 17.7 | 17.4 | 5.6 | 30.1 | 0.23% | 1.0 |
| R | 33.6 | 22.3 | 21.1 | 43.9 | 0% | 1.5 |

Differences between groups:

| Δ | 2.5% | 97.5% | Prob to reject null | | Effect size |
| --- | --- | --- | --- | --- | --- |
|  |  |  | $\Delta>0$ | $\Delta=0$ |  |
| 15.8 | -1.4 | 32.0 | 4% | 91% | 3.4 |

**Late early rise in Explicit Eye:**

| group | $\mu$ | $\sigma$ | 2.5% | 97.5% | Prob to reject null ($\Delta>0)$ | Effect size |
| --- | --- | --- | --- | --- | --- | --- |
| NR | 24.7 | 14.8 | 13.2 | 35.6 | 0% | 1.6 |
| R | 44.2 | 5.1 | 41.0 | 47.4 | 0% | 8.6 |

Differences between groups:

| Δ | 2.5% | 97.5% | Prob to reject null | | Effect size |
| --- | --- | --- | --- | --- | --- |
|  |  |  | $\Delta>0$ | $\Delta=0$ |  |
| 19.5 | 7.9 | 31.4 | 0.05% | 99% | 6.3 |

**End of adaptation in Explicit Eye:**

| group | $\mu$ | $\sigma$ | 2.5% | 97.5% | Prob to reject null ($\Delta>0)$ | Effect size |
| --- | --- | --- | --- | --- | --- | --- |
| NR | 18.9 | 14.4 | 11.1 | 27.3 | 0% | 1.3 |
| R | 36.8 | 9.6 | 31.7 | 41.7 | 0% | 3.8 |

Differences between groups:

| Δ | 2.5% | 97.5% | Prob to reject null | | Effect size |
| --- | --- | --- | --- | --- | --- |
|  |  |  | $\Delta>0$ | $\Delta=0$ |  |
| 18.0 | 8.3 | 27 | 0.05% | 99% | .6 |

**Initial rise in Explicit Report:**

|  | $\mu$ | $\sigma$ | 2.5% | 97.5% | Prob to reject null ($\Delta>0)$ | Effect size |
| --- | --- | --- | --- | --- | --- | --- |
| R | 32.4 | 23.1 | 18.7 | 43.9 | 0% | 1.4 |

**Late early rise in Explicit Report:**

|  | $\mu$ | $\sigma$ | 2.5% | 97.5% | Prob to reject null ($\Delta>0)$ | Effect size |
| --- | --- | --- | --- | --- | --- | --- |
| R | 44.3 | 7.1 | 40.8 | 48.6 | 0% | 6.2 |

**End of adaptation in Explicit Report:**

|  | $\mu$ | $\sigma$ | 2.5% | 97.5% | Prob to reject null ($\Delta>0)$ | Effect size |
| --- | --- | --- | --- | --- | --- | --- |
| R | 37.7 | 9.1 | 33.5 | 41.5 | 0% | 4.1 |

**Report explicit - eye explicit within a group:**

|  | Δ | 2.5% | 97.5% | Prob to reject null (Δ=0) | Effect size |
| --- | --- | --- | --- | --- | --- |
| R | -0.02 | -1.6 | 1.5 | 0% | 0 |

**Report implicit - eye implicit within a group:**

|  | Δ | 2.5% | 97.5% | Prob to reject null (Δ=0) | Effect size |
| --- | --- | --- | --- | --- | --- |
| R | -0.3 | -1.9 | 1.4 | 0% | -0.3 |

**Eye Implicit - After effect within a group:**

| Group | Δ | 2.5% | 97.5% | Prob to reject null (Δ=0) | Effect size |
| --- | --- | --- | --- | --- | --- |
| NR | 19.6 | 10.8 | 28.4 | 100% | 1.7 |
| R | 3.4 | -2.2 | 9.3 | 29% | 0.5 |

Differences between groups:

| Δ | 2.5% | 97.5% | Prob to reject null (R>NR) | Effect size |
| --- | --- | --- | --- | --- |
| -16.2 | -26.5 | -5.8 | 0% | -1.8 |

**after effect Vs implicit eye: correlation**

| Group | Mean correlation | 2.5% | 97.5% | Prob to reject null (r>0.2) |
| --- | --- | --- | --- | --- |
| NR | 0.67 | 0.36 | 0.79 | 0.1% |
| R | 0.75 | 0.49 | 0.83 | 0% |

**Experiment 2:**

**Implicit Eye – Catch:**

| cluster | Δ | $\sigma$ | 2.5% | 97.5% | Prob to reject null ($\Delta=0$) | Effect size |
| --- | --- | --- | --- | --- | --- | --- |
| all | 5.4 | 8.1 | 2.7 | 8.1 | 62% | 0.6 |
| Match Low | -0.5 | 4.5 | -5.6 | 4.0 | 5% | -0.1 |
| No Match | 13.3 | 4.7 | 10.0 | 16.7 | 100% | 2.8 |
| Match High | -0.3 | 4.3 | -3.5 | 2.8 | 0.3% | -0.06 |

**Explicit Eye – Catch:**

| cluster | Δ | $\sigma$ | 2.5% | 97.5% | Prob to reject null (Δ=0) | Effect size |
| --- | --- | --- | --- | --- | --- | --- |
| all subjects | -5.6 | 8.3 | -8.0 | -3.2 | 69% | -0.7 |
| Match Low | -0.1 | 5.0 | -3.5 | 3.9 | 1% | -0.02 |
| No Match | -13.5 | 5.0 | -16.5 | -10.6 | 100% | -2.7 |
| Match High | 0.5 | 4.3 | -1.8 | 2.8 | 0% | 0.1 |

**Initial rise in Hand-Target Difference:**

| group | $\mu$ | $\sigma$ | 2.5% | 97.5% | Prob to reject null ($\Delta>0)$ | Effect size |
| --- | --- | --- | --- | --- | --- | --- |
| all | 25.2 | 21.0 | 17.9 | 32.4 | 0% | 1.2 |
| Match Low | 3.6 | 13.7 | -7.8 | 14.7 | 25% | 0.3 |
| No Match | 33.5 | 16.0 | 22.8 | 42.5 | 0% | 2.1 |
| Match High | 31.8 | 20.0 | 18.0 | 44.4 | 0% | 1.6 |

Differences between clusters:

| $x_{1}$ | $x_{2}$ | Δ | 2.5% | 97.5% | Prob to reject null | | Effect size |
| --- | --- | --- | --- | --- | --- | --- | --- |
|  |  |  |  |  | $\Delta>0$ | $\Delta=0$ |  |
| Match High | Match Low | 28.1 | 10.6 | 45.4 | 0.06% | 100% | 6.8 |
| No Match | Match Low | 29.8 | 14.5 | 44.4 | 0% | 100% | 7.8 |
| Match High | No Match | -1.7 | -18.1 | 14.9 | 59% | 55.4% | -0.4 |

**Late early rise in Hand-Target Difference:**

| group | $\mu$ | $\sigma$ | 2.5% | 97.5% | Prob to reject null ($\Delta>0)$ | Effect size |
| --- | --- | --- | --- | --- | --- | --- |
| all | 34.6 | 26.6 | 29.7 | 32.3 | 0% | 2.1 |
| Match Low | 12.7 | 14.5 | 4.3 | 22.2 | 0.1% | 0.9 |
| No Match | 39.8 | 10.2 | 34.0 | 45.1 | 0% | 3.9 |
| Match High | 45.4 | 4.7 | 41.0 | 49.7 | 0% | 9.6 |

Differences between clusters:

| $x_{1}$ | $x_{2}$ | Δ | 2.5% | 97.5% | Prob to reject null | | Effect size |
| --- | --- | --- | --- | --- | --- | --- | --- |
|  |  |  |  |  | $\Delta>0$ | $\Delta=0$ |  |
| Match High | Match Low | 32.7 | 22.3 | 42.3 | 0% | 100% | 10.9 |
| No Match | Match Low | 27.0 | 15.7 | 37.2 | 0% | 100% | 7.9 |
| Match High | No Match | 5.6 | -1.3 | 12.7 | 6% | 57% | 2.0 |

**End of adaptation in Hand-Target Difference:**

| group | $\mu$ | $\sigma$ | 2.5% | 97.5% | Prob to reject null ($\Delta>0)$ | Effect size |
| --- | --- | --- | --- | --- | --- | --- |
| all | 40.2 | 14.0 | 35.9 | 44.4 | 0% | 2.99 |
| Match Low | 22.7 | 14.4 | 14.3 | 31.0 | 0% | 1.5 |
| No Match | 44.5 | 7.4 | 39.4 | 49.3 | 0% | 6.0 |
| Match High | 48.7 | 5.6 | 43.8 | 53.5 | 0% | 8.7 |

Differences between clusters:

| $x_{1}$ | $x_{2}$ | Δ | 2.5% | 97.5% | Prob to reject null | | Effect size |
| --- | --- | --- | --- | --- | --- | --- | --- |
|  |  |  |  |  | $\Delta>0$ | $\Delta=0$ |  |
| Match High | Match Low | 26.1 | 16.3 | 35.8 | 0% | 100% | 8.5 |
| No Match | Match Low | 21.8 | 12.0 | 31.4 | 0% | 100% | 6.9 |
| Match High | No Match | 4.2 | -2.6 | 11.2 | 11.2% | 42% | 1.6 |

**Initial rise in Explicit Eye:**

| group | $\mu$ | $\sigma$ | 2.5% | 97.5% | Prob to reject null ($\Delta>0)$ | Effect size |
| --- | --- | --- | --- | --- | --- | --- |
| all | 20.2 | 18.7 | 14.2 | 26.5 | 0% | 1.1 |
| Match Low | 3.0 | 9.4 | -4.7 | 11.9 | 23% | 0.3 |
| No Match | 22.6 | 148 | 14.6 | 30.4 | 0% | 1.5 |
| Match High | 31.0 | 19.8 | 19.0 | 43.0 | 0% | 1.6 |

Differences between clusters:

| $x_{1}$ | $x_{2}$ | Δ | 2.5% | 97.5% | Prob to reject null | | Effect size |
| --- | --- | --- | --- | --- | --- | --- | --- |
|  |  |  |  |  | $\Delta>0$ | $\Delta=0$ |  |
| Match High | Match Low | 28.1 | 13.0 | 42.4 | 0% | 100% | 7.2 |
| No Match | Match Low | 19.6 | 7.7 | 30.5 | 0.1% | 99% | 5.5 |
| Match High | No Match | 8.5 | -6.0 | 23.0 | 12% | 73% | 2.1 |

**Late early rise in Explicit Eye:**

| group | $\mu$ | $\sigma$ | 2.5% | 97.5% | Prob to reject null ($\Delta>0)$ | Effect size |
| --- | --- | --- | --- | --- | --- | --- |
| all | 25.0 | 17.9 | 19.9 | 30.1 | 0% | 1.4 |
| Match Low | 5.8 | 11.2 | -1.2 | 14.6 | 6% | 0.5 |
| No Match | 24.1 | 13.9 | 17.5 | 31.3 | 0% | 1.7 |
| Match High | 41.6 | 8.9 | 36.1 | 46.9 | 0% | 4.7 |

Differences between clusters:

| $x_{1}$ | $x_{2}$ | Δ | 2.5% | 97.5% | Prob to reject null | | Effect size |
| --- | --- | --- | --- | --- | --- | --- | --- |
|  |  |  |  |  | $\Delta>0$ | $\Delta=0$ |  |
| Match High | Match Low | 35.8 | 25.5 | 44.8 | 0% | 100% | 11.3 |
| No Match | Match Low | 18.3 | 7.5 | 28.4 | 0% | 99% | 5.1 |
| Match High | No Match | 17.4 | 8.5 | 26.0 | 0% | 100% | 5.1 |

**End of adaptation in Explicit Eye:**

| group | $\mu$ | $\sigma$ | 2.5% | 97.5% | Prob to reject null ($\Delta>0)$ | Effect size |
| --- | --- | --- | --- | --- | --- | --- |
| all | 23.3 | 16.8 | 18.3 | 28.6 | 0% | 1.4 |
| Match Low | 7.1 | 14.1 | -2.0 | 21.0 | 6% | 0.5 |
| No Match | 21.5 | 12.0 | 14.8 | 28.1 | 0% | 1.8 |
| Match High | 38.8 | 9.0 | 34.3 | 43.3 | 0% | 4.3 |

Differences between clusters:

| $x_{1}$ | $x_{2}$ | Δ | 2.5% | 97.5% | Prob to reject null | | Effect size |
| --- | --- | --- | --- | --- | --- | --- | --- |
|  |  |  |  |  | $\Delta>0$ | $\Delta=0$ |  |
| Match High | Match Low | 31.7 | 17.1 | 42.0 | 0% | 100% | 9.4 |
| No Match | Match Low | 14.3 | -0.7 | 25.7 | 3% | 92% | 4.0 |
| Match High | No Match | 17.3 | 9.3 | 25.2 | 0% | 100% | 5.3 |

**End of adaptation in Implicit Eye:**

| group | $\mu$ | $\sigma$ | 2.5% | 97.5% | Prob to reject null ($\Delta>0)$ | Effect size |
| --- | --- | --- | --- | --- | --- | --- |
| all | 17.0 | 12.4 | 12.2 | 21.4 | 0% | 1.4 |
| Match Low | 16.1 | 16.7 | 1.2 | 27.0 | 2% | 1.0 |
| No Match | 22.5 | 9.5 | 16.7 | 28.1 | 0% | 2.3 |
| Match High | 10.6 | 8.3 | 5.0 | 16.2 | 0% | 1.3 |

Differences between clusters:

| $x_{1}$ | $x_{2}$ | Δ | 2.5% | 97.5% | Prob to reject null | | Effect size |
| --- | --- | --- | --- | --- | --- | --- | --- |
|  |  |  |  |  | $\Delta<0$ | $\Delta=0$ |  |
| Match High | Match Low | -5.5 | -18.0 | 10.1 | 20% | 65% | 1.0 |
| No Match | Match Low | 6.4 | -6.1 | 22.4 | 83% | 59% | 2.4 |
| Match High | No Match | -11.9 | -19.8 | -3.7 | 0% | 95% | 1.3 |

**Decrease in RT:**

| group | $\mu$ | $\sigma$ | 2.5% | 97.5% | Prob to reject null ($\Delta>0)$ | Effect size |
| --- | --- | --- | --- | --- | --- | --- |
| Match Low | 450 | 820 | -189 | 1182 | 8% | 0.5 |
| No Match | 272 | 497 | -162 | 733 | 11% | 0.5 |
| Match High | 758 | 1066 | 73 | 1537 | 1% | 0.7 |

**RT in baseline:**

| group | $\mu$ | $\sigma$ | 2.5% | 97.5% | Prob to reject null ($\Delta>0)$ | Effect size |
| --- | --- | --- | --- | --- | --- | --- |
| Match Low | 831 | 483 | 634 | 1091 | 0% | 1.7 |
| No Match | 676 | 301 | 574 | 798 | 0% | 2.2 |
| Match High | 1080 | 627 | 833 | 1376 | 0% | 1.7 |

Differences between clusters:

| $x_{1}$ | $x_{2}$ | Δ | 2.5% | 97.5% | Prob to reject null | | Effect size |
| --- | --- | --- | --- | --- | --- | --- | --- |
|  |  |  |  |  | $\Delta<0$ | $\Delta>0$ |  |
| Match High | Match Low | 249 | -104 | 610 | 8% | 91% | 1.7 |
| No Match | Match Low | -155 | -433 | 79 | 89% | 10% | 2.2 |
| Match High | No Match | 404 | 130 | 725 | 0.1% | 100% | 1.7 |
